# FibroSURE as a noninvasive marker of liver fibrosis and inflammation in chronic hepatitis B

**DOI:** 10.1186/1471-230X-14-118

**Published:** 2014-07-03

**Authors:** Marija Zeremski, Rositsa B Dimova, Samantha Benjamin, Jessy Makeyeva, Rhonda K Yantiss, Maya Gambarin-Gelwan, Andrew H Talal

**Affiliations:** 1 Division of Gastroenterology and Hepatology, Department of Medicine, Weill Cornell Medical College, New York, NY, USA; 2 Department of Biostatistics, State University of New York at Buffalo, Buffalo, NY, USA; 3 Department of Pathology and Laboratory Medicine, Weill Cornell Medical College, New York, NY, USA; 4 Gastroenterology and Nutrition Service, Memorial Sloan-Kettering Cancer Center, New York, NY, USA; 5 Division of Gastroenterology, Hepatology and Nutrition, Department of Medicine, State University of New York at Buffalo, Buffalo, NY, USA

**Keywords:** Hepatitis B virus, Liver biopsy replacement, Liver fibrosis assessment, Liver histology

## Abstract

**Background:**

Noninvasive markers of liver fibrosis have not been extensively studied in patients with chronic hepatitis B virus (HBV) infection. Our aim was to evaluate the capacity of FibroSURE, one of the two noninvasive fibrosis indices commercially available in the United States, to identify HBV infected patients with moderate to severe fibrosis.

**Methods:**

Forty-five patients who underwent liver biopsy at a single tertiary care center were prospectively enrolled and had FibroSURE performed within an average interval of 11 days of the biopsy.

**Results:**

Of the 45 patients, 40% were Asian, 40% were African American, and 13% were Caucasian; 27% were co-infected with HIV and 67% had no or mild fibrosis. We found FibroSURE to have moderate capacity to discriminate between patients with moderate to high fibrosis and those with no to mild fibrosis (area under receiver operating characteristic [AUROC] curve = 0.77; 95% confidence interval [CI] [0.61, 0.92]). When we combined the fibrosis score determined by FibroSURE with aspartate aminotransferase (AST) measurements and HIV co-infection status, the discriminatory ability significantly improved reaching an AUROC of 0.90 (95% CI [0.80, 1.00]). FibroSURE also had a good ability to differentiate patients with no or mild from those with moderate to high inflammation (AUROC = 0.83; 95% CI [0.71, 0.95]).

**Conclusions:**

FibroSURE in combination with AST levels has an excellent capacity to identify moderate to high fibrosis stages in chronic HBV-infected patients. These data suggest that FibroSURE may be a useful substitute for liver biopsy in chronic HBV infection.

## Background

Chronic hepatitis B virus (HBV) infection affects 350 to 400 million people worldwide [1,2]. The disease can progress to cirrhosis and hepatocellular carcinoma in approximately 15% to 40% of patients. The long-term complications are more frequent in patients with active viral replication as well as in those with significant intrahepatic inflammation and fibrosis [3]. Therefore, assessment of liver pathology is very important clinically as it identifies patients most likely to benefit from antiviral treatment.

Currently, the “gold standard” for the assessment of hepatic fibrosis is liver biopsy. Liver biopsy is recommended by the most recent AASLD guidelines on HBV in order to determine the need for antiviral therapy for those with normal liver enzymes who meet treatment criteria based on the viral load [4,5]. However, the procedure has several drawbacks including morbidity, mortality, significant sampling error, and high cost. It is associated with marked inter- and intra- observer/pathologist variability, and the use of different scoring systems to assess the degree of intrahepatic inflammation and fibrosis further complicates comparisons between pathologists. These limitations are important issues, particularly when patients seek a second opinion [6]. Finally, the invasive nature of the biopsy makes it unsuitable for serial hepatic sampling. In an attempt to overcome these limitations, several indices that incorporate parameters associated with inflammation or fibrogenesis, such as FibroTest/FibroSURE [7,8], Forns’ index [9], or the aspartate aminotransferase (AST)/platelet ratio index [10], have been proposed as noninvasive alternatives. FibroSURE (Laboratory Corporation of America (LabCorp), Burlington, NC) and FIBROSpect II (Prometheus Laboratories, San Diego, CA) are the only two commercially available noninvasive indices used in the United States. Both of these tests have been developed and extensively validated in patients with hepatitis C [11,12], but their utility in hepatitis B has not been adequately evaluated. In this study, we prospectively assessed the performance characteristics of FibroSURE as a noninvasive test for measuring liver inflammation and fibrosis in patients with chronic hepatitis B infection.

## Methods

### Patients

All HBV-infected patients who underwent a liver biopsy at our institution between 2005 and 2012 were prospectively approached and offered participation in this study. We enrolled 45 HBV-infected patients, 12 (26.7%) of whom were co-infected with HIV. Patients with HCV co-infection and other co-existing liver diseases were excluded. The study was approved by the Weill Cornell Medical College institutional review board and was performed in accordance with the Declaration of Helsinki. After signing an informed consent, each patient’s blood was drawn and sent to LabCorp for performance of the FibroSURE. Alanine aminotransferase (ALT) and AST levels, as well as HBV DNA measurements, were extracted from the electronic medical record and were performed, on average, within 1 month (ALT and AST), or within 2 months (HBV DNA), of the liver biopsy or FibroSURE. All liver biopsies were evaluated according to the Scheuer classification [13] by the same hepatopathologist (RKY).

### Statistical analysis

Statistical analysis was performed using SAS (SAS Institute Inc., Cary, NC, USA) and R (http://www.r-project.org/). Aminotransferase levels were logarithmically transformed for the analysis. Associations between categorical variables were assessed through Fisher’s exact test and logistic regression. For continuous variables, comparisons between groups were performed using Wilcoxon rank-sum or Kruskal-Wallis tests. Diagnostic abilities of FibroSURE were expressed through the estimated area under the receiver operating characteristic (AUROC) curve and by calculation of the respective sensitivity, specificity, positive and negative predictive values estimated for appropriate cut-off values. The AUROC was estimated by the trapezoidal rule and represents the probability that a randomly selected pair of subjects will be classified correctly. In order to estimate the linear combination of the available variables, which detects fibrosis stage and inflammation grade, we used logistic regression. Comparison of the ROC curves was based on the methods described by DeLong *et al.*[14]. The significance level in all tests (two-sided) was set to 0.05.

## Results

### Patients’ characteristics

A total of 45 patients with chronic HBV infection, 12 of whom were HIV/HBV co-infected, were included in this study. Patients’ mean age was 42.4 years, 58% were male, 40% were Asian, 40% were African American, and 13% were Caucasian (Table 1). Among the twelve HIV/HBV co-infected patients, 83.3% were African American and 16.7% were Caucasian. Ten patients, nine of whom were HIV co-infected, were on treatment for chronic HBV infection during the time of the liver biopsy/FibroSURE. One patient was taking Lamivudine, two were on Tenofovir, four patients were on Truvada, and three patients, one of whom was HBV mono-infected, were on Entecavir. Most of the study participants had mild liver disease: 53% had necroinflammation less than or equal to grade 1 and 67% had stage 0–1 fibrosis. In addition, 55.6% of patients had ALT levels that were in the normal range (0 – 45 U/L). Only 8.9% of patients had ALT values greater than two times the upper limit of normal.

**Table 1:** Patients characteristics

**Parameter**		**Patients (N = 45)**
**Mean (SD) age, years**		42.4 (12.15)
**Gender**	Male	26 (58%)
	Female	19 (42%)
**Ethnicity**	Caucasian	6 (13%)
	African American	18 (40%)
	Asian	18 (40%)
	Hispanic	1 (2%)
	Indian	2 (4%)
**Grade**	0	4 (9%)
	1	20 (44%)
	2	14 (31%)
	3	6 (13%)
	4	1 (2%)
**Stage**	0	18 (40%)
	1	12 (27%)
	2	8 (18%)
	3	4 (9%)
	4	3 (7%)
**HIV co-infected**		12 (27%)
**Mean ALT (U/L) (range)**		53 (10–240)
**Mean AST (U/L) (range)**		46 (13–302)
**Mean HBV DNA (IU/ml) (range)**		33,476,858 (<20-200,000,000)
**Positive HBeAg**		19 (42%)

ALT, AST, and HBV DNA measurements were not available for one HBV-infected patient.

*Abbreviations*: ALT alanine aminotransferase, AST aspartate aminotransferase, SD standard deviation, HBV hepatitis B virus.

FibroSURE test was performed an average of 11 days preceding or after the liver biopsy. However, in the majority of patients (62%), it was performed on the same day as the liver biopsy.

HIV/HBV co-infected patients had significantly higher fibrosis levels, as estimated by both liver biopsy (p = 0.0099) and FibroSURE (p = 0.0425), compared to HBV mono-infected patients. Although the histologic grade of intrahepatic necroinflammation did not differ significantly depending upon type of infection, co-infected patients had significantly higher FibroSURE inflammatory scores compared to mono-infected patients (p = 0.0341). Of note, HBV/HIV co-infected patients had significantly higher ALT levels compared to HBV mono-infected patients (p = 0.017).

### FibroSURE as a predictor of liver fibrosis

**Figure 1:**
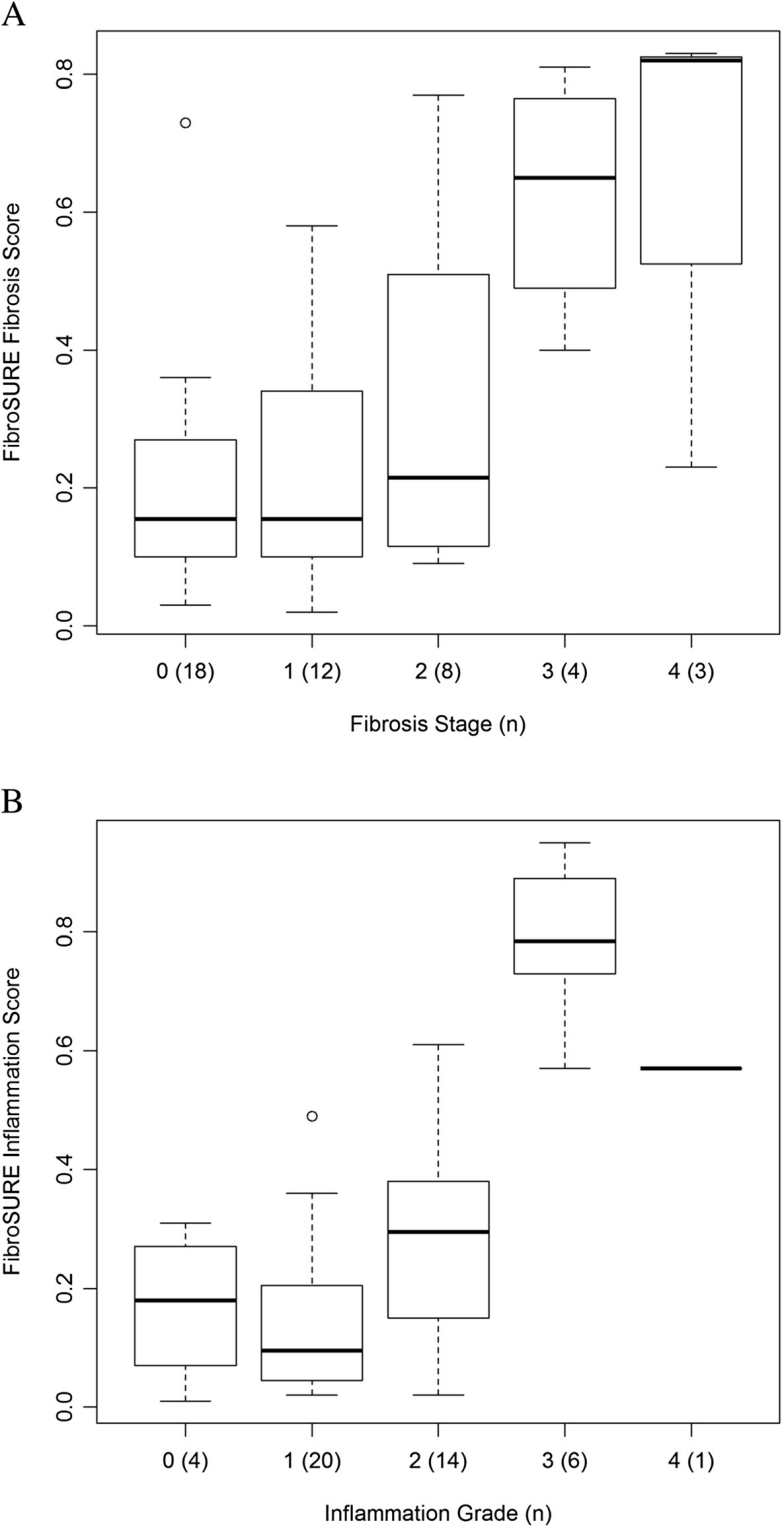
**Boxplots illustrating the distribution of FibroSURE fibrosis scores stratified by fibrosis stage (A), and FibroSURE inflammation scores stratified by inflammatory grade (B).** The number of people in each category is listed in parenthesis adjacent to the fibrosis stage. Please note that only seven patients had advanced fibrosis and inflammation (stages/grades 3 or 4).

As expected, values obtained on FibroSURE were significantly higher in patients with moderate to severe fibrosis (stage ≥ 2) compared to those with mild or no fibrosis (stage ≤ 1) (p = 0.0158) (Figure 1A; Table 2). Advanced fibrosis was also associated with HIV co-infection (p = 0.0156) and grade of inflammation (p = 0.0066). Patients with advanced fibrosis had significantly higher ALT (p = 0.0067) and AST (p = 0.035) levels, but only based on univariable logistic regression analysis (Table 2).

**Table 2:** Logistic regression analysis on moderate to severe stage of fibrosis

		**Stage > =2 (n = 15) n (%) or Mean (SD)**	**Stage < 2 (n = 30) n (%) or Mean (SD)**			**Univariable logistic regression**	Multivariable logistic regression***
**Variable**				**OR**	**95% CI**	**p-value**	**p-value**
Age		46.87 (13.93)	40.17 (10.72)	1.05	0.993; 1.110	0.089	
Gender, N (%)	Male	11 (42.31)	15 (57.69)	2.75	0.713; 10.605	0.1418	0.0641
	Female*	4 (21.05)	15 (78.95)				
Race, N (%)	Caucasian*	1 (16.67)	5 (83.33)			0.2845	
	AA	9 (50.00)	9(50.00)	5	0.483; 51.769		
	Asian	4 (22.22)	14 (77.78)	1.429	0.127; 16.025		
	Other	1 (33.33)	2 (66.67)	2.5	0.1; 62.602		
Diagnosis	HBV	7 (21.21)	26 (78.79)	0.135	0.031; 0.581	0.0072**	0.0156**
	HIV/HBV*	8 (66.67)	4 (33.33)				
Inflammation	Grade > 1	12 (57.14)	9 (42.86)	9.333	2.110; 41.278	0.0032**	0.0066**
	Grade < =1*	3 (12.50)	21 (87.50)				
FibroSURE		0.462 (0.297)	0.212 (0.167)	3.015^§^	1.426; 6.374	0.0039**	0.0158**
log_10_ ALT (U/L)		1.81 (0.25)	1.52 (0.28)	3.314^§^	1.393; 7.883	0.0067**	
log_10_ AST (U/L)		1.70 (0.21)	1.51 (0.25)	2.455^§^	1.065; 5.658	0.0350**	
log_10_ HBV DNA (IU/ml)		5.24 (2.98)	4.60 (2.39)	1.304^§^	0.674; 2.520	0.4303	

*Reference group for the reported odds ratios.

**p-value < 0.05.

***Based on the best selected model.

^§^OR of 1 SD increase.

*Abbreviations*: ALT alanine aminotransferase, AST aspartate aminotransferase, HBV hepatitis B virus, SD standard deviation, OR odds ratio, CI confidence interval, AA African-American, HIV human immunodefiency virus.

**Figure 2:**
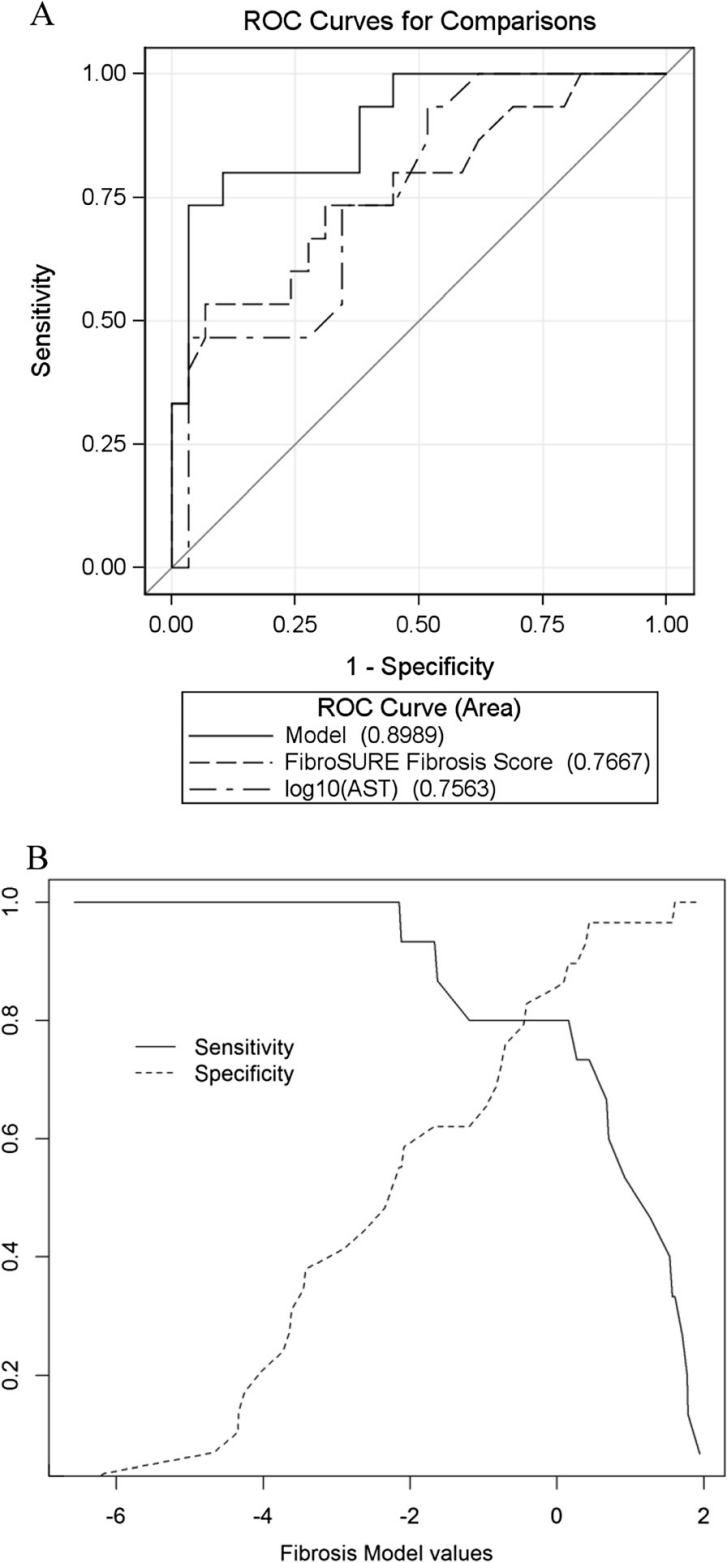
**Receiver operating characteristic (ROC) curves of the combined model (as described in Results), FibroSURE and log**_**10**_**(AST) for the detection of moderate to severe fibrosis (stage ≥ 2).** Values of the area under ROC curves for the individual variables are indicated in parenthesis at the bottom of the image **(A)**. Sensitivity and specificity of the model score for a range of cut-off values **(B)**.

When we evaluated the ability of FibroSURE to discriminate between patients with mild or no fibrosis (stage ≤ 1) and those with moderate to severe fibrosis (stage ≥ 2), the calculated area under the ROC curve was 0.77 (95% confidence interval [CI] 0.61, 0.92). ALT and AST measurements had similar discriminatory abilities (AUROC = 0.79; 95% CI: 0.65, 0.92; p = 0.8096 and AUROC = 0.76; 95% CI: 0.61, 0.90; p = 0.9064, respectively). For example, a cut-off value of FibroSURE ≥ 0.49 resulted in sensitivity of 0.47, specificity of 0.93, positive predictive value (PPV) of 0.78 and negative predictive value (NPV) of 0.77. However, the combination of FibroSURE with AST and HIV co-infection status improved its capacity to identify patients with moderate to severe (stage ≥ 2) fibrosis. Based on logistic regression, the following linear score:

$$\begin{array}{ll} 1.65 & +2.26\times \mathrm{FibroSURE}‒19.28\\ & \times 1_{\left\{\mathrm{if}\;\mathrm{HBV}\;\mathrm{mono}‒\mathrm{infected}\right\}}‒1.07\times \mathrm{lo}g_{10}\left(\mathrm{AST}\right)+10.93\\ & \times 1_{\left\{\mathrm{if}\;\mathrm{HBV}\;\mathrm{mono}‒\mathrm{infected}\right\}}\mathrm{lo}g_{10}\left(\mathrm{AST}\right) \end{array}$$

had AUROC of 0.90 (95% CI [0.80, 1.00]) (Figure 2A), which was significantly better than either FibroSURE (p = 0.0156) or AST (p = 0.0450) values individually. When we evaluated the performance characteristics of the model, we found that a cut-off score of 0.44 or higher resulted in sensitivity of 0.73, specificity of 0.97, PPV of 0.92 and NPV of 0.88. A cut-off score of −2.15 had sensitivity of 1, specificity of 0.55, PPV of 0.54 and NPV of 1 to identify patients with moderate to high fibrosis (Figure 2B).

### FibroSURE as a predictor of liver inflammation

**Figure 3:**
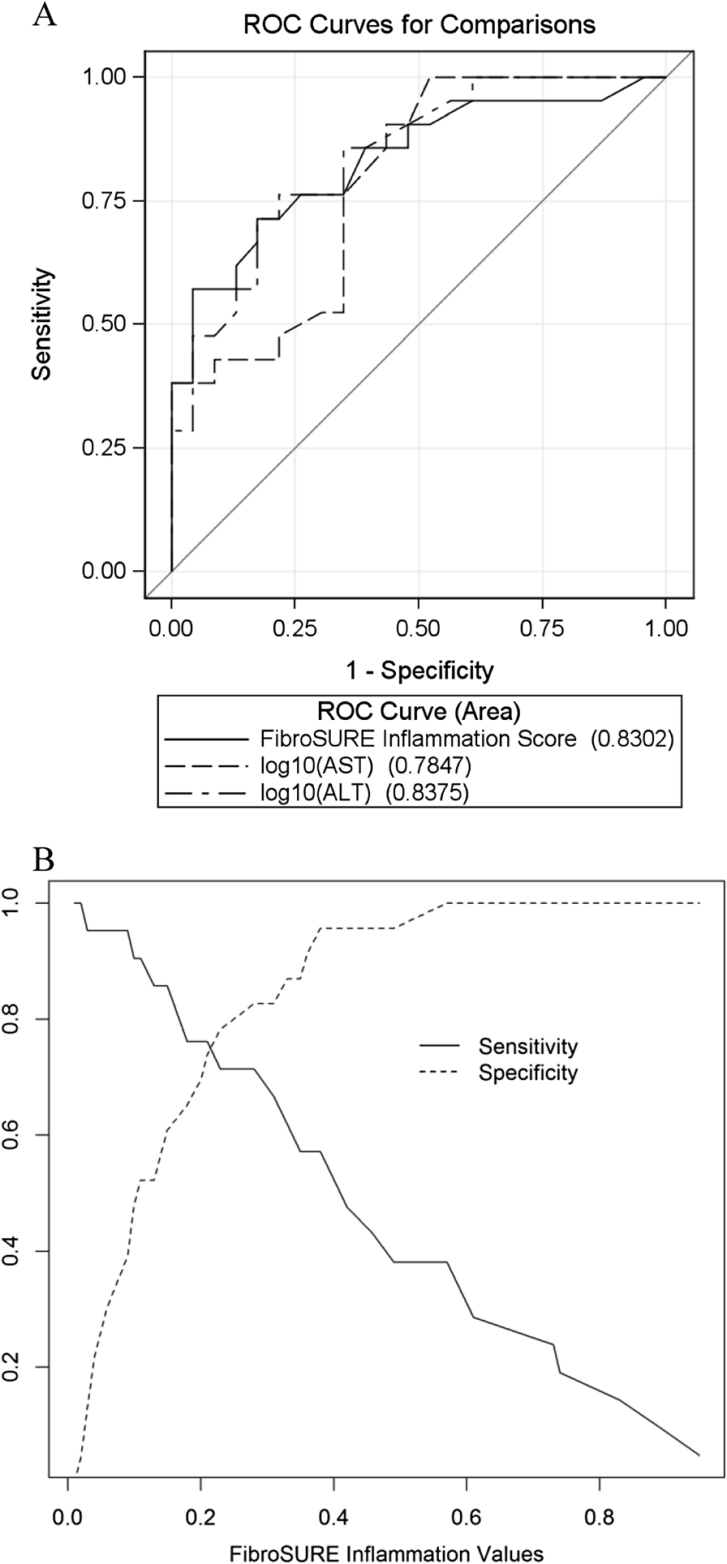
**Receiver operating characteristic (ROC) curves of the FibroSURE inflammation score, log**_**10**_**(AST) and log**_**10**_**(ALT) for the detection of moderate to severe inflammation (grade ≥ 2) (A).** Sensitivity and specificity of the FibroSURE inflammation score for a range of cut-off values **(B)**.

FibroSURE inflammatory scores were significantly higher in patients with higher intrahepatic inflammation compared to those with mild or no inflammation (stage ≤ 1) (p = 0.0003) (Figure 1B). Based on univariable logistic regression analysis, advanced inflammation was also associated with male gender (p = 0.0228), higher stages of fibrosis (p = 0.0032), ALT levels (p = 0.0014), AST levels (p = 0.0047), and HBV DNA (p = 0.0135).FibroSURE had good ability to differentiate patients with mild or no inflammation (grade ≤ 1) from those with moderate to high inflammation (grade ≥ 2); AUROC = 0.83 (95% CI [0.71, 0.95]) (Figure 3A). Using a cut-off value of 0.52 for FibroSURE inflammation score, we obtained sensitivity of 0.38, specificity of 1, PPV of 1 and NPV of 0.639 (Figure 3B). However, the ability of FibroSURE to indicate moderate to high inflammation in chronic HBV patients was not significantly higher compared to ALT (AUROC = 0.84; 95% CI: 0.72, 0.95; p = 0.8191) or AST (AUROC = 0.78; 95% CI: 0.65, 0.92; p = 0.4132) measurements individually (Figure 3A). The addition of AST, HBV DNA measurements, or male sex to the FibroSURE inflammation score did not improve its discriminatory ability. As ALT is a component of the FibroSURE score, evaluation of the combination of ALT and inflammation as assessed by FibroSURE would not improve the diagnostic performance.

## Discussion

Unlike HCV, in HBV, treatment endpoints include biochemical responses, as indicated by normalization of ALT, and histological responses, as indicated by reduced necroinflammation and reversion of fibrosis [2]. Although liver biopsy has been considered the “gold standard” for the assessment of intrahepatic pathology, the procedure has significant limitations including invasiveness, high cost and poor reproducibility [6]. In the last decade, several non-invasive indices have been developed as liver biopsy alternatives. While indices, such as FibroSURE and Fibrospect, have largely been developed and validated in patients with chronic HCV infection, their use in HBV-infected patients has not been nearly as extensive. Given the therapeutic importance of necroinflammatory activity and fibrosis in HBV infection, a noninvasive index such as FibroSURE might have an important role in the management of the infection.

In the United States, FibroSURE is one of the two commercially available noninvasive indices of hepatic fibrosis and inflammation. FibroSURE is a multicomponent index comprised of six serum markers including α2-macroglobulin, haptoglobin, apolipoprotein A-1, total bilirubin, γ-glutamyltransferase, and ALT that are combined with patients’ age and gender to calculate separate scores for fibrosis and necroinflammatory activity. It is currently marketed for liver histology assessment in chronic HCV-infected patients, and only a few studies evaluated the utility of this test in chronic HBV infection [15-20]. However, in all of these studies, as opposed to performing FibroSURE in a commercial laboratory where the reproducibility of the test is closely monitored and subject to performance measures, serum marker measurements in the prior studies were performed in local laboratories and calculation of the fibrosis score was performed individually by study authors. To our knowledge, the commercially available FibroSURE test has not been evaluated previously in chronic HBV-infected patients from the United States.

Our data showed that FibroSURE had reasonable ability to discriminate between HBV-infected patients with moderate to high fibrosis and those with mild or no fibrosis. Moreover, the performance of FibroSURE test in our HBV-infected patients was very similar to results previously published for patients infected with HCV [11]. The addition of AST and HIV co-infection status to the FibroSURE score significantly improved its discriminatory capacity, with an AUROC of 0.90, making the combination of these three parameters an excellent index for identification of HBV-infected patients with advanced fibrosis. As a marker of intrahepatic inflammation, FibroSURE showed good ability to discriminate between patients with moderate to high inflammation and those with mild or no inflammation. However, its discriminatory ability did not differ significantly from either ALT or AST individually.

Excellent performance characteristics are only the initial attribute required for the adaptation in the clinic of any diagnostic test. In addition, tests must be conveniently obtainable and readily interpretable in the clinical setting with a high degree of reproducibility as demonstrated through laboratory performance characteristics. Reproducibility is particularly important for serial application of a test, such as might be required of FibroSURE if it were to be used as a benchmark for determining treatment outcome or if it were to be performed repeatedly to assess progression of inflammation or fibrosis. We [21] and others [22] have previously shown that the commercially available FibroSURE has the utility to identify fibrosis progression in patients with HCV. Whether FibroSURE has similar prognostic significance in HBV infection remains to be determined.

A limitation of this study is the small sample size. Although all HBV-infected patients who underwent a liver biopsy at our institution between 2005 and 2012 were consecutively approached, their number was small and not all who were approached agreed to study participation. However, the racial diversity of our subjects is representative of the U.S. population of HBV-infected individuals. In contradistinction to other countries, HBV infection prevalence in the United States is estimated to be ~2% among Asians and 0.73% among non-Hispanic Blacks, the ethnicities with the highest HBV prevalence in the U.S. [23]. These two groups account for 39% and 31% of individuals, respectively, in the U.S. with chronic HBV infection. The ethnicity of patients in this study (40% Asian, 40% Black) is reflective of that found in the general U.S. population and can be considered a strength of our study population. An additional limitation of the study is the heterogeneous patient population consisting of unequal numbers of HBV mono-infected and HIV/HBV co-infected patients.

## Conclusions

In chronic HBV infection, liver biopsy is usually performed in patients with active disease in order to identify those who might be expected to benefit the most from treatment. However, many patients are reluctant to undergo the procedure due to its invasiveness and high costs. Our study shows that performance of FibroSURE in combination with AST levels can be a good substitute for liver biopsy for estimation of hepatic fibrosis in HBV-infected patients. The test can be performed on one tube of blood easily obtained at the point of care. Based upon these results, we believe that the FibroSURE can be used to identify patients in whom HBV treatment should be considered.

## Competing interests

This study was funded in part through the Fellows Research Program sponsored by Bristol-Myers Squibb and Laboratory Corporation of America for performance of the Fibrosure test.

## Authors’ contributions

MZ and AHT designed the study. MZ, SB, JM, and MG-G acquired the data. MZ, RBD, RKY, and AHT analyzed and interpreted the data. RBD performed statistical analysis of the data. MZ and AHT wrote the manuscript. All authors read and approved the final version of the manuscript.

## Pre-publication history

The pre-publication history for this paper can be accessed here:

http://www.biomedcentral.com/1471-230X/14/118/prepub
